# Hydrothermal Carbonization of Cellulose with Ammonium Sulfate and Thiourea for the Production of Supercapacitor Carbon

**DOI:** 10.3390/polym15234478

**Published:** 2023-11-21

**Authors:** Chang Liu, Kun Wang, Yuhan Du, Yaqi Shan, Peigao Duan, Naveed Ramzan

**Affiliations:** 1 Shaanxi Key Laboratory of Energy Chemical Process Intensification, School of Chemical Engineering and Technology, Xi’an Jiaotong University, Xi’an 710049, China; chang509891@stu.xjtu.edu.cn (C.L.); kun-wang@stu.xjtu.edu.cn (K.W.); yqshan@xjtu.edu.cn (Y.S.); 2 P-129 Staff Colony, University of Engineering and Technology, Lahore 39161, Pakistan

**Keywords:** hydrothermal carbonization, cellulose, ammonium sulfate, thiourea, doping, supercapacitor carbon

## Abstract

Doping with heteroatoms is the main method used to enhance energy storage with carbon materials, and polyatomic doping is one of the main challenges. Hydrothermal carbonization of cellulose was performed at 240 °C for 1 h. Ammonium sulfate and thiourea dopants were selected as the sources of inorganic nitrogen and organic nitrogen in the preparation of supercapacitor carbon. The effects of boric acid on the properties of the resulting hydrochar after KOH activation were examined. The results showed that the proportion of functional groups and the specific surface area of the activated hydrochar were reduced by the addition of boric acid, and the formation of micropores was inhibited. The hydrochar obtained from the reaction of cellulose and organic nitrogen compounds had a better pore size distribution and electrochemical properties after activation. The largest specific surface area (952.27 m^2^/g) was obtained when thiourea was used as the sole dopant. In a three-electrode system, the specific capacitance of the activated hydrochar reached 235.8 F/g at a current density of 1 A/g. After 20,000 charging and discharging cycles at a current density of 10 A/g, the capacitance retention rate was 99.96%. Therefore, this study showed that supercapacitor carbon with good electrochemical properties was obtained by the direct reactions of cellulose with organic nitrogen compounds.

## 1. Introduction

Fossil fuels have large impacts on human life activities and industrial production. According to the World Energy Council, the world will need twice as much energy in 2050 as it currently consumes [1]. Therefore, there is a need for new energy sources to replace fossil fuels. Replacing conventional energy systems by implementing renewable energy systems is considered a crucial step toward sustainable development [2,3].

Renewable energy requires an environmentally friendly and efficient energy storage device. The energy technologies available include solar energy, wind energy, tidal energy, geothermal energy, etc. These new energy sources involve the effects of factors such as geographical location and climatic conditions [4,5,6]. After decades of development, supercapacitors have attracted extensive attention due to their high power densities, fast charge and discharge speeds, and long-term cycling stabilities compared with traditional secondary batteries [7]. These outstanding properties make supercapacitors promising carriers for use in portable electronics, hybrid electric vehicles, etc., and in building renewable energy systems [8,9,10,11].

However, a significant feature currently limiting the use of supercapacitors is their low energy densities [12,13,14]. Some researchers have noted that the use of new materials, such as nanoscale metal oxides [15,16], conductive polymers [17], and heteroatom-doped (B, N, S, P, O) carbon, could improve energy densities [18,19,20,21,22]. For example, nitrogen enrichment is an effective way to achieve N doping of supercapacitor carbon materials. The nitrogen functional groups in supercapacitor carbon materials improve the electron transfer rate and stability, reduce the equivalent series resistance, and thus improve the electrochemical performance of the material [23,24]. Many reports have also pointed out that supercapacitor carbon materials have considerable advantages in many industrial applications due to their large specific surface areas and porous structures. High porosity is an important parameter because it provides adsorption sites for ion storage and promotes ion transport [25,26,27,28,29,30].

There are many raw materials available for the preparation of supercapacitor carbon materials, among which biomass has attracted interest due to several advantages, including high yields, renewability, little pollution, zero carbon dioxide emissions, and so on. At present, the conversion of biomass to supercapacitor carbon materials is mainly achieved via thermochemical methods [31,32,33,34,35], among which hydrothermal carbonization (HTC) shows much promise because it uses wet biomass directly and produces substantial economic benefits [36]. During the HTC process, hydrogen bonding between the water molecules is weakened, and both the ionization constant and the concentration of H^+^ in the liquid phase are increased [37]. Additionally, the pH of the liquid phase is also increased by the addition of organic acids, which promote the hydrolysis of oligosaccharides and other substances, thereby enabling the formation of oxygen-containing functional groups on the surface of the resulting hydrochar [38]. However, the doping sources or the organic acids produced during the HTC treatment of biomass are limited, and additional organic acids must be added.

There have been many reports of adding additives to improve the performance of carbon materials prepared in various ways. For example, Susanti et al. added a small amount of citric acid to the HTC of salacca peel to increase the specific surface area of the carbon material [39]. Deng et al. added an N source to activated carbon to prepare electrodes with high spectral surface areas and dense nanopores [40]. Guo et al. removed heavy metals through HTC by adding urea as a nitrogen source [41]. Huang et al. prepared carbon materials for supercapacitor use by adding melamine as a nitrogen source during the HTC of bamboo shoot shells [42]. Wu et al. used urea as a nitrogen source during the HTC of carboxymethyl cellulose to prepare microporous, spherical carbonaceous adsorbents (CSn) used for carbon dioxide capture [43].

The doping source has a great influence on the electrochemical properties of the material. Among the doping sources, boric acid is widely used as a source of B, whichimproves the electrochemical performance of supercapacitor carbon, and doping with B and N further improves the electrochemical properties of the material. Zhao et al. realized an inexpensive and simple preparation of B, N-doped porous C by using freeze-dried chitosan-boric acid aerogel beads followed by tube furnace carbonization. B doping significantly affected the distribution of N species in the carbon material. The resulting doped carbon presented good wettability and supported the diffusion of electrolytes and transport [44]. Liang et al. developed an efficient strategy for carbonizing chitosan in the presence of boric acid, and the resulting carbon showed a high specific capacitance, a large operating voltage, and excellent cycling stability for use in supercapacitors [45].

In terms of polyatomic doping to prepare supercapacitors, Shan et al. used ammonium sulfate and cellulose hydrothermal carbonization to prepare supercapacitor carbon materials codoped with nitrogen and sulfur, which improved the specific capacitance and other properties of the material, but the capacitance of the material showed a relatively low retention rate of only 76.55% [46]. Djandja et al. prepared a supercapacitor carbon material with higher capacitance retention by adding pyridine/pyrrole as the nitrogen source [47].

Therefore, in the present study, the HTC of cellulose was examined with the addition of an inorganic N source (ammonium sulfate) or an organic N source (thiourea) for the production of supercapacitor carbon. The effects of B addition on the specific surface areas, pore structures, and electrochemical performance of the resulting carbon materials prepared by adding organic and inorganic nitrogen sources were elaborated in detail. Finally, the electrochemical capabilities of the activated carbon materials were characterized by cyclic voltammetry (CV), galvanostatic charge–discharge (GCD) tests, electrochemical impedance spectroscopy (EIS), and cycling lifetime measurements.

## 2. Materials and Methods

### 2.1. Materials

α-Cellulose powder (CL) was purchased from Shanghai Macklin Biochemical Technology Co., Ltd. (Shanghai, China). Thiourea (TU), ammonium sulfate (AS), and boric acid (BA) were purchased from Tianjin Damao Chemical Reagent Factory (Tianjin, China). Deionized water was prepared in the laboratory, and it showed a conductivity of less than 10 μs/cm. Potassium hydroxide (KOH) and acetylene black BP2000 were purchased from Tianjin Kermel Chemical Reagent Co., Ltd. (Tianjin, China). The polytetrafluoroethylene (PTFE) binder was provided by Shanghai McLean Biochemical Technology Co., Ltd. (Shanghai, China).

HTC was performed with a mechanical reactor MSG100-P5-T3-SS1-SV-R (Anhui CHEMᴺ Instrument Co., Ltd., Hefei, China) with a volume of 100 mL. The reactor was equipped with a stirrer. The heating power of the reactor was 1.1 kW. The rated temperature and pressure of the reactor were 300 °C and 20.7 MPa, respectively.

### 2.2. HTC

Five grams of CL, 0.5 g of TU or AS, 0.5 g of BA (when needed), and 40 mL of deionized water were loaded into a 100 mL beaker and mixed uniformly to form a paste. This paste was transferred into a 100 mL stainless-steel reactor and sealed. The switch was turned on to start the heating. Approximately 30 min later (depending on the reaction temperature), the temperature inside the reactor had reached 240 °C, and the reaction time was counted at this point. After a 1 h reaction time, the reactor was placed into a water bath. Once the temperature inside the reactor reached room temperature, it was removed from the water bath. If gaseous products had formed in the reactor, the valve was opened to release the gas products first. The solid (hydrochar) and the aqueous phase were transferred into a Büchner funnel for separation. The hydrochar was dried in an oven at 105 °C for 12 h. The yield of hydrochar was equal to the mass of the hydrochar divided by the total mass of the material initially added into the reactor. The hydrochar (HC) produced from the HTC of CL, CL+TU, CL+AS, CL+TU+BA, and CL+AS+BA were named HC-CL, HC-CL-TU, HC-CL-AS, HC-CL-TU-BA, and HC-CL-AS-BA, respectively.

### 2.3. Activation of HC

HC (0.5 g) and 1.5 g of KOH were loaded into a 250 mL beaker, and 200 mL deionized water was added into the beaker and subjected to ultrasonication for 20 min. The HC was soaked in the KOH solution for 24 h. The soaked HC was separated with a Büchner funnel and dried at 105 °C for 12 h. The soaked HC was loaded into a porcelain boat and placed into a tube furnace, which was heated to 800 °C at a heating rate of 10 °C/min under an Ar environment with a flow rate of 20 mL/min and then kept at 800 °C for 1 h. After activation, the activated hydrochar (AHC) was cooled to room temperature under an Ar atmosphere, washed with deionized water until the pH of the filtrate reached 7, and dried at 105 °C for 12 h. The activated hydrochar (AHC) produced HC-CL, HC-CL-TU, HC-CL-AS, HC-CL-TU-BA, and HC-CL-AS-BA, which were named AHC-CL, AHC-CL-TU, AHC-CL-AS, AHC-CL-TU, and AHC-CL-AS-AS-BA, respectively.

### 2.4. Characterization of HC and AHC

The specific surface areas (SSAs) and pore size distributions of the AHCs were determined by N_2_ adsorption-desorption experiments run at 77 K with a JW-BK100 system (Beijing JWGB Instruments Co., Ltd., Beijing, China). The morphologies and microstructures of the samples were determined with a Gemini SEM 500 scanning electron microscope (Carl Zeiss AG, Oberkochen, Germany). X-ray diffraction was performed with an XRD-6100 diffractometer (Shimadzu Corporation, Nakagyo-ku, Kyoto, Japan). Infrared spectra were obtained with a Nicolet iS50 spectrophotometer (Thermo Fisher Scientific, Waltham, MA, USA). Thermogravimetric analyses were performed with a STA449F5 system (Netzsch, Selb, Germany). X-ray photoelectron spectroscopy (XPS) was performed with an ESCALAB 250Xi (Thermo Scientific, USA). Elemental analyses were obtained with a Vario EL cube (Elementar Americas Inc., Ronkonkoma, NY, USA).

### 2.5. Electrochemical Performance of AHC

The electrochemical capabilities of the individual electrodes were evaluated with a model CHI1030B electrochemical workstation (Shanghai Chenhua Instrument Co., Ltd., Shanghai, China). A three-electrode cell was used under an ambient atmosphere, in which a platinum sheet served as the counter electrode and Hg/HgO served as the reference electrode.

AHC, acetylene black, and PTFE were mixed with a mass ratio of 16:3:1 and dissolved in ethanol. The mixture was subjected to sonication at a power of 120 W for 20 min and dried at 105 °C for 5 h to completely remove the ethanol. The dried mixture was pressed into flakes with uniform masses under a pressure of 2 MPa and cut into circular flakes with diameters of 10 mm. Two pieces of nickel foam were cut into circular slices with diameters of 15 mm. The 10 mm diameter electrode discs and a nickel sheet were placed between the two nickel foam discs and extruded with a tablet press at a pressure of 4 MPa to make the working electrode. The electrolyte used in the tests was 6 mol/L KOH. The electrochemical studies included cyclic voltammetry (CV) with scan rates ranging from 10 to 200 mV/s for CV curves, galvanostatic charge/discharge (GCD) at current densities between 1 and 20 A/g, and electrochemical impedance spectroscopy (EIS) at frequencies between 0.01 Hz and 100 kHz. In the land cell test program, the cycling stability was examined over 20,000 cycles at 10 A/g. The determination of the specific capacitance based on three-electrode system GCD technology is expressed by Formula (1) [48]:

(1) $$C=\frac{I\Delta t}{m\Delta V}$$

where C is the gravimetric capacitance of the electrode (F/g), I is the discharge current (A), Δt is the discharge time (s), m is the mass of the carbon (g), and ΔV is the voltage range (V vs. Hg/HgO).

In the two-electrode test, the electrode preparation method and test equipment were consistent with the above three-electrode test procedures. Two electrodes of equal weight were prepared for testing. One electrode served as the count/reference electrode, while the other served as the test electrode. The electrolyte used in the experiment was a KOH solution with a concentration of 6 mol/L. Electrochemical testing included two main techniques: CV and GCD. In the CV test, scan rates ranged from 10 to 200 mV/s, while the GCD test involved current densities ranging from 0.5 to 20 A/g. The specific capacitance, energy density, and power density were determined using Formulas (2)–(4), respectively [48]:

(2) $$C_{s}=\frac{2I\Delta t}{m\Delta V}$$

(3) $$E=\frac{C_{s}{\left(∆V\right)}^{2}}{3.6\times 2}$$

(4) $$P=3600(\frac{E}{\Delta V})$$

where C_s_ is the specific capacitance (F/g) of the symmetrical capacitor, I is the discharge current (A), Δt is the discharge time (s), m is the mass of the carbon(g), ΔV is the voltage range (V vs. Hg/HgO), E is the specific energy (Wh/kg), and P is the specific power (W/kg).

## 3. Results and Discussion

### 3.1. Yield of the HC and Elemental Analyses of the HC and AHC

**Figure 1:**
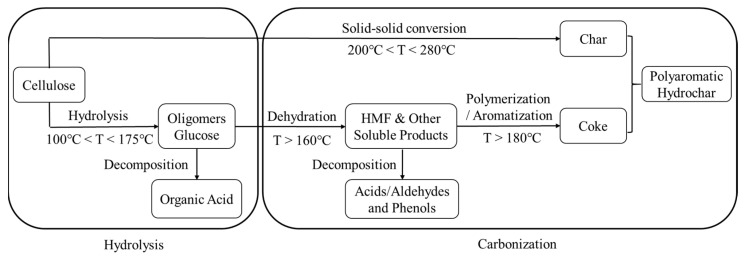
The reaction path for CL carbonization under hydrothermal conditions.

The HC yield for direct HTC of CL was 52.8 wt%. Whether AS or TU was added, the HC yield from CL was slightly increased and remained at approximately 53.5 wt%. The reactions occurred between CL and AS or TU, and the addition of BA reduced the hydrochar yield slightly to approximately 53 wt%. Figure 1 shows the reaction path for CL carbonization under hydrothermal conditions [49]. The aqueous solution of BA was acidic, which was not optimal for the carbonization of CL or its hydrolysates. In addition, acidic conditions also facilitated the hydrolysis of oligosaccharides into organic acids and the decomposition of HMF into acids/aldehydes and phenols, thus reducing the hydrochar yield.

Table 1 presents the elemental compositions of the HC and AHC produced from various feedstocks. Compared with CL, the hydrochar obtained from HTC of CL exhibited a higher C content (71.14 wt%) and lower H (4.35 wt%) and O (24.07 wt%) contents. This suggested the carbonization process of CL, as illustrated in Figure 1. This observation was supported by the low H/C and O/C ratios of the hydrochar obtained via the HTC of CL. The incorporation of AS or TU into CL has been found to enhance the HTC of CL because the resulting hydrochar exhibited a higher C content than that prepared from pure CL. Compared with that from CL alone, AS and TU produced higher hydrogen (H) contents in the resulting hydrochar. This suggested that the addition of AS and TU increased the H content of the resulting hydrochar, which had a higher H content after adding AS and TU. The reaction between CL and AS or TU resulted in higher contents of N and S in the hydrochar. Furthermore, the higher the concentration of N and S in the additive, the greater the concentration of N and S in the resulting hydrochar. It is worth mentioning that the S content of hydrochar produced from the reaction of CL and AS was only 0.35 wt%, whereas the hydrochar obtained from TU had a sulfur content of 4.54 wt%. This suggested that organic sulfur was more likely to react with CL. The hydrochar showed increased contents of C, H, N, and S from AS or TU. Consequently, there was a corresponding decrease in the oxygen (O) content. The inclusion of BA facilitated the reaction between CL and AS or TU, resulting in enhanced N and S contents in the resulting hydrochar. The H/C ratio of the hydrochar decreased, indicating that BS promoted the carbonization processes of CL with AS or TU. Additionally, BA also engaged in the reaction and augmented the oxygen content in hydrochar with a low boron content.

Since activation was carried out at high temperatures and in an inert atmosphere, all of the activated hydrochar had a higher C content and lower H and O contents than hydrochar. The H/C ratios of the activated hydrochars were as low as 0.02. The contents of N and S remaining after activation of the hydrochar also showed different degrees of loss, so the N and S contents in the activated hydrochar were lower than those in the hydrochar.

### 3.2. FT-IR and XRD Analyses of HC

**Figure 2:**
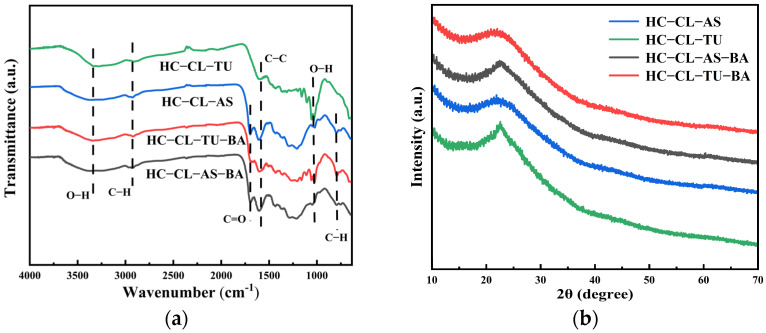
(**a**) FTIR spectra of HC-CL-TU, HC-CL-AS, HC-CL-TU-BA and HC-CL-AS-BA; (**b**) XRD of HC-CL-TU, HC-CL-AS, HC-CL-TU-BA and HC-CL-AS-BA.

To determine the thermal decomposition properties, the functional groups of the hydrochar were analyzed with FTIR. Figure 2a shows that absorption bands were observed for HC-CL-TU, HC-CL-AS, HC-CL-TU-BA, and HC-CL-AS-BA within the range 2800–3000 cm^−1^, and they were attributed to the vibrational modes of aliphatic C–H bonds [50]. The absorption band observed at 1650 cm^−1^ was attributed to the stretching vibration of C=O of the carboxylic acid (-COOH) functional group in the amino acid [51]. The absorption band at 1540 cm^−1^ was attributed to the stretching vibrations of C=C double bonds within the aromatic rings. The absorption band at 1450 cm^−1^ corresponded to the stretching of the C–C bonds in aromatic rings, suggesting the formation of a carbon material with a higher degree of carbonization. The absorption band at 1040 cm^−1^ was attributed to a stretching vibration of the C–O–R bonds in ethers. This suggested that as the carbonization process progressed, the degree of carbonization increased, resulting in the formation of O–H bonds. The absorption band at 780 cm^−1^ was attributed to the vibrational motions of the C–H bonds in aromatic compounds.

X-ray diffraction (XRD) was employed to determine the crystallinity of the porous carbon. As depicted in Figure 2b, a prominent peak at 22.4° corresponded to the (002) planes [52]. Figure 2b shows that the elimination of boron led to a proportional increase in the width of the peak, suggesting a higher disorder. It is evident that carbonization without boron led to increased disorder. This increase was attributed to an alteration in the distribution of pore sizes that were formed during carbonization. The obtained result is consistent with the pore size analysis.

### 3.3. BET Analysis of the AHC

**Figure 3:**
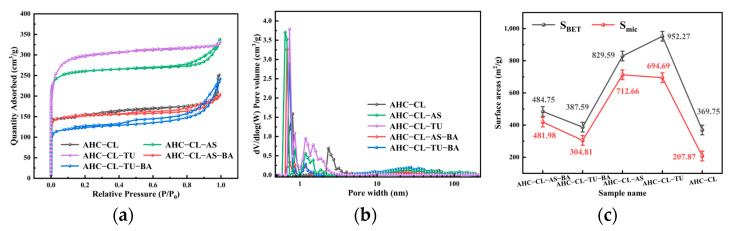
(**a**) Adsorption/desorption curves for AHC-CL, AHC-CL-AS, AHC-CL-TU, AHC-CL-AS-BA, and AHC-CL-TU-BA in a N_2_ environment; (**b**) pore size distribution curves for AHC-CL, AHC-CL-AS, AHC-CL-TU, AHC-CL-AS-BA, and AHC-CL-TU-BA; (**c**) specific surface areas and micropore areas of AHC-CL, AHC-CL-AS, AHC-CL-TU, AHC-CL-AS-BA, and AHC-CL-TU-BA.

The porosities of AHC-CL, AHC-CL-AS, AHC-CL-TU, AHC-CL-AS-BA, and AHC-CL-TU-BA were examined with N_2_ adsorption/desorption at a temperature of 77 K. Figure 3a illustrates that none of the isotherms for AHC-CL-TU exhibited noticeable hysteresis loops, indicating type-I behavior. However, AHC-CL-AS-BA, AHC-CL-TU-BA, and AHC-CL-AS showed obvious hysteresis loops. At lower relative pressures (P/P_0_ < 0.1), there were significant increases in nitrogen uptake, suggesting that the as-prepared AHCs were microporous. When the relative pressure (P/P_0_) was increased to 0.2, there was a gradual increase in the uptake of nitrogen. This was attributed, at least in part, to the presence of micro/mesoporosity. Compared to AHC-CL-AS-BA and AHC-CL-TU-BA, AHC-CL-AS and AHC-CL-TU exhibited significantly higher adsorption capacities, suggesting that AHC-CL-AS and AHC-CL-TU had higher SSAs. The limited pore structure generation during HTC may be attributed to the presence of boron, which led to a decrease in the reduction in SSA. Additionally, it is evident that the impact of boron doping on the doping of TU was considerably greater than its effect on AS. The inclusion of BA resulted in a decrease in the specific surface area of the AHC doped with TU and AS, and the reduction in the specific surface area of the experimental group doped with TU was more obvious.

**Figure 4:**
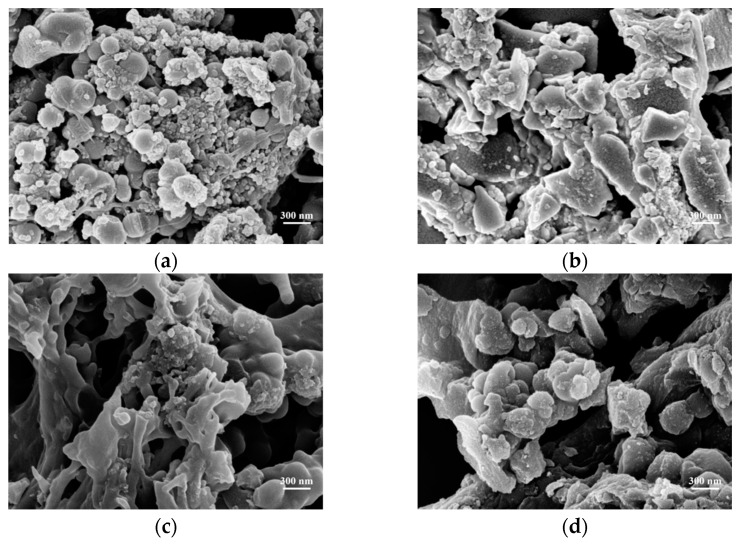
SEM images of (**a**) AHC-CL-AS; (**b**) AHC-CL-AS-BA; (**c**) AHC-CL-TU and (**d**) AHC-CL-TU-BA.

Figure 3b clearly demonstrates a notable decrease in the number of micropores formed in the AHC after boron doping. This observation provides further evidence that boron occupied the pores of the AHC, consequently leading to a reduction in the SSA of the AHC. As depicted in Figure 4, the SEM images revealed that AHC-CL-TU and AHC-CL-AS exhibited more pores than AHC-CL-TU-BA and AHC-CL-AS-BA.

As indicated in Figure 3c, the SSA and micropore area of the AHCs were suppressed when boron was added, especially when BA and TU were cocarbonized, and the SSA and micropore area of the resulting AHCs were significantly lower. When only TU was added, the SSA of the AHC reached 952.27 m^2^/g, and the micropore area reached 694.69 m^2^/g. With only CL+AS, the SSA of the AHC was 829.59 m^2^/g, and the micropore area was 712.66 m^2^/g. When boron was added, the specific surface area of AHC-CL-AS-BA was reduced to 484.76 m^2^/g, and the micropore area was reduced to 418.99 m^2^/g; the specific surface area of AHC-CL-TU-BA was reduced to 397.59 m^2^/g, and its micropore area was reduced to 304.81 m^2^/g. This may be because the added BA occupied the micropore sites during HTC, resulting in a decrease in the micropore area of the AHC sample. After the introduction of boron, noticeable changes were observed in both the SSA and the micropore area. Among the different groups, the most significant impact was observed for the group doped with TU. When boron was not doped, the specific surface area of the AHC with added AS was significantly smaller than that of the AHC with added TU. However, the prepared micropore area remained almost the same. This observation suggested that the addition of AS produced more micropores. The addition of AS to the AHC demonstrated a heightened capacity to generate micropores while simultaneously mitigating the interference caused by boron.

### 3.4. XPS Analysis of the AHC

**Figure 5:**
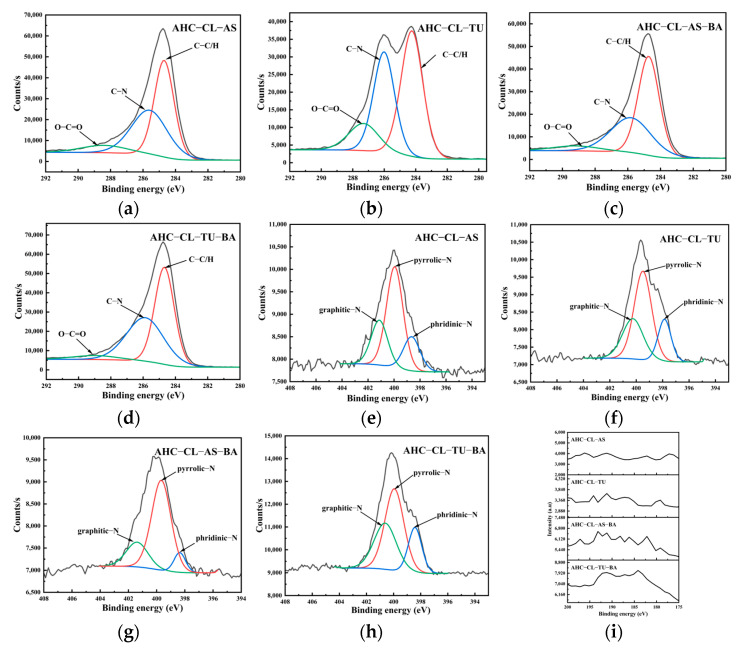
XPS spectra of AHC-CL-AS, AHC-CL-TU, AHC-CL-AS-BA, and AHC-CL-TU-BA; (**a**–**d**) C1s, (**e**–**h**) N1 s and (**i**) B1s high-resolution spectra.

XPS of the AHC was used to determine the chemical states of the elements on the surface of the AHC. Figure 5 shows the results. The peaks at 286.1 eV and 287.6 eV represent C–N and O–C=O groups, respectively. Compared with the control group (AS), the experimental group doped with TU exhibited a significant increase in the peak intensity for C–N/C–O. Additionally, the experimental group doped with TU exhibited a significantly higher C=O peak intensity than the group doped with AS. This showed that, compared with AS doping, the AHC doped with TU underwent decarboxylation during HTC. It was demonstrated that the incorporation of TU increased the amount of oxygen-containing functional groups (OFGs) in synthesized AHCs. This, in turn, increased the wettability of the AHC pores and facilitated the entry of ions into the pores.

The primary peaks at 399.1 eV and 398.3 eV were attributed to pyrrole-N and pyridine-N, respectively. The increased pyrrole-N content in the AHC led to the generation of additional electrochemically active sites, consequently enhancing the faradaic capacitance by changing the electron distribution on the surface of the AHC. As indicated in Table 1, the TU-doped AHC exhibited a higher N content, but the pyrrole-N peak intensity for AHC-CL-TU was lower than that of AHC-CL-AS, suggesting that not all of the nitrogen was converted into pyrrole-N during HTC. Instead, it was present in the AHC as a different N form. AHC-CL-AS had more pyrrole-N, which indicates that this carbon may exhibit better charge transfer performance and electron supply performance.

It is evident from the data presented in Table 2 that the mass fractions of elements in AHCs, as determined through XPS analyses, were consistent with those in Table 1. Compared to AHC-CL-AS-BA (0.39 wt%), the B content in AHC-CL-TU-BA (0.80 wt%) was higher, suggesting that the use of TU as a nitrogen source was more effective in polyatomic doping. The conditions favored B doping. Combined with Figure 5i, the AHC-CL-TU-BA and AHC-CL-AS-BA samples exhibited noticeable increases in the peak intensities, suggesting successful doping of B into the AHCs.

When both nitrogen and boron were simultaneously introduced, the C–N peak intensity for AHC-CL-TU-BA was significantly higher than that for AHC-CL-AS-BA. This suggested that the B-assisted TU synergistic carbonization process increased the formation of C–N. Additionally, the peak intensity for O–C=O after the B-assisted TU synergistic carbonization process was higher than that of the B-assisted AS synergistic carbonization process. This indicated that the B-assisted TU synergistic carbonization process facilitated decarboxylation during the HTC process. During carbonization, assisted by the synergistic effect of CL/TU and B addition, the increased pyrrole-N content generated additional electrochemically active sites on the surfaces of the AHCs. Consequently, this change in electron distribution increased the faradaic capacitance of the AHC. The intensity of the pyrrole-N peak in AHC-CL-AS-BA was comparable to that observed for AHC-CL-TU-BA. Although AHC-CL-AS-BA and AHC-CL-TU-BA exhibited similar N contents, it is noteworthy that the intensity of the pyridine-N peak for AHC-CL-TU-BA was significantly greater than that for AHC-CL-AS-BA. A significant reduction in the C–N peak intensity for the experimental group doped with TU was observed after the addition of B and N. These results indicated that the inclusion of B impeded the formation of C−N species. Moreover, after adding B, the C=O peak intensity also decreased significantly. This showed that the addition of B and TU facilitated carbonization, which inhibited decarboxylation. This reduced the OFGs in the AHC and reduced the wettabilities of the AHC pores, thus preventing ions from entering the pores. After adding B as a dopant, the intensity of the pyrrole-N peak for AHC decreased. As indicated in Table 1, the N contents for the two AHCs were similar, indicating that not all N was converted into pyrrole-N during the HTC or activation process but existed in the AHC as other forms of N. The pyrrole-N content of AHC-CL-TU was higher, indicating that this sample may have better charge transfer performance and electron supply performance.

In the course of incorporating AS as the N source, the introduction of B as a dopant resulted in a notable decrease in the intensity of the C–N peak in the AS-doped experimental group. It is evident that the inclusion of B impeded the formation of C–N bonds during HTC or activation. Additionally, the inclusion of B resulted in a decrease in the C=O peak intensity. These findings indicated that the introduction of B and TU for the synergistic carbonization process hindered decarboxylation, thereby impeding the carbonization process. This was meant to decrease the OFGs present in the AHCs, diminish the wettabilities of the AHC pores, and consequently impede the infiltration of ions into the pores. After doping with B, the intensity of the peak for pyrrole-N in AHC remained consistent. As indicated in Table 1, the N content of AHC-CL-AS-BA was considerably greater than that of AHC-CL-AS, suggesting that not all of the nitrogen was converted into pyrrole-N during HTC and/or activation processes. Instead, some nitrogen was present in the AHC in a different form.

### 3.5. Electrochemical Analysis of the AHC

**Figure 6:**
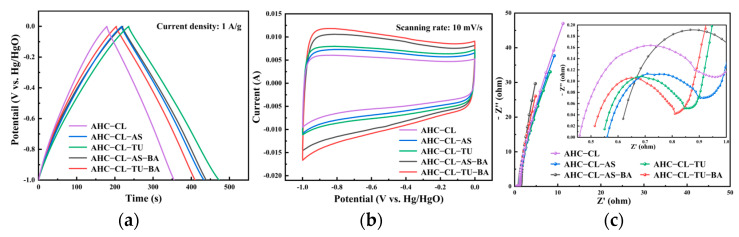
Electrochemical performance data determined with a three-electrode system using 6 mol/L potassium hydroxide solution as the electrolyte; (**a**) GCD curves for AHC-CL-TU, AHC-CL-AS, AHC-CL-TU-BA, and AHC-CL-AS-BA at a current density of 1 A/g; (**b**) CV curves for AHC-CL-TU, AHC-CL-AS, AHC-CL-TU-BA, and AHC-CL-AS-BA at a scan rate of 10 mV/s and (**c**) Nyquist spectra of AHC-CL-TU, AHC-CL-AS, AHC-CL-TU-BA, and AHC-CL-AS-BA.

A three-electrode system was used to study the electrochemical performance of AHC in a 6 mol/L KOH electrolyte solution with cyclic voltammetry (CV), galvanostatic charge–discharge (GCD), and EIS. In the three-electrode test, the obtained test results were relative to the reference electrode of Hg/HgO. To investigate the impact of B-assisted TU and AS synergistically carbonized cellulose on the electrochemical performance of the AHC, GCD and CV studies were conducted with the AHC-CL-TU, AHC-CL-AS, AHC-CL-TU-BA, and AHC-CL-AS-BA samples. As depicted in Figure 6a, the AHC exhibited symmetrical triangular distributions, which suggest favorable electric double-layer capacitance and electrochemical reversibility. The AHC-CL-TU exhibited longer charging and discharging times. At a current density of 1 A/g, the mass-specific capacitance of AHC-CL-TU was 236.25 F/g. The aforementioned observation suggested that AHC-CL-TU has a greater capacity for energy storage.

Figure 6b displays the CV curves of AHC-CL-TU, AHC-CL-AS, AHC-CL-TU-BA, and AHC-CL-AS-BA obtained at a scan rate of 10 mV/s. The CV curves of AHC-CL-TU-BA and AHC-CL-AS-BA exhibited larger quasirectangular areas with slight protrusion and minimal deformation. This suggested that the electric double-layer capacitor sample contained a small amount of oxygen, which was attributed to the redox reactions of the surface functional groups during the rapid charge and discharge processes. AHC-CL-TU and AHC-CL-AS exhibited more regular equirectangular shapes, suggesting that the electrolyte ions underwent rapid ionic reactions with these two samples.

Figure 6c shows the EIS data for AHC-CL-TU, AHC-CL-AS, AHC-CL-TU-BA, and AHC-CL-AS-BA. The Nyquist plots of the four sample groups (AHC-CL-TU, AHC-CL-AS, AHC-CL-TU-BA, and AHC-CL-AS-BA) exhibited small quasisemicircles at higher frequencies. Additionally, they exhibited pronounced linearity in the low-frequency range, a distinctive feature commonly observed in carbon-based materials. All of them exhibit favorable electrical conductivity and ion diffusion characteristics. The semicircle observed on the real axis provides an approximation of the charge transfer resistance. By observing the semicircular cross-section connected to the x-axis, the following conclusions can be drawn. Regardless of the inclusion or exclusion of B, the charge transfer resistance for the experimental group utilizing TU as the nitrogen source was lower. With the addition of BA, the charge transfer resistance of the experimental group using TU as the nitrogen source decreased, and the charge transfer resistance of the experimental group using AS as the nitrogen source increased and was even higher than that of the experimental group without heteroatom doping (AHC-CL). This showed that for the different nitrogen sources, the addition of BA had different effects on the material properties. The Nyquist curves for AHC-CL-TU, AHC-CL-AS, AHC-CL-TU-BA, and AHC-CL-AS-BA exhibited nearly linear behavior in the low-frequency region. It is evident that the addition of AS/TU as the nitrogen source resulted in a decrease in the slope of the material in the low-frequency region. Furthermore, the experimental group utilizing AS as the nitrogen source exhibited a slightly higher slope than the experimental group using TU as the nitrogen source. After the addition of BA, the slopes of AHC-CL-TU-BA and AHC-CL-AS-BA both exhibited increases, with the latter slope showing a slightly higher increment.

**Figure 7:**
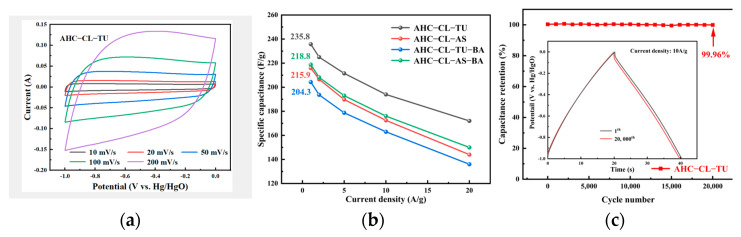
Electrochemical performance data determined in a three-electrode system using 6 mol/L potassium hydroxide as the electrolyte; (**a**) CV curves determined for AHC-CL-TU at scan rates of 20 to 200 mV/s; (**b**) histogram showing the specific capacitance at flow densities of 1, 2, 5, 10, and 20 A/g; (**c**) based on AHC-CL-TU, supercapacitors were tested for stability with 20,000 cycles at a current density of 10 A/g.

Figure 7a shows the CV curves for AHC-CL-TU at various scan rates ranging from 20 to 200 mV/s. It is evident that at lower scan rates, the data should have quasirectangular shapes with minor protrusions and small deformations. This suggested double electric layer phenomena, which were attributed to the redox reactions of surface functional groups during discharge of the sample. Figure 7b shows the specific capacitances of AHC-CL-TU, AHC-CL-AS, AHC-CL-TU-BA, and AHC-CL-AS-BA in the three-electrode system. Using Formula (1), the specific capacitance of the three-electrode system can be calculated. At a current density of 1 A/g, AHC-CL-TU had the highest specific capacitance, at 235.8 F/g, which was much higher than those of AHC-CL-AS (215.9 F/g), AHC-CL-TU-BA (204.3 F/g), and AHC-CL-AS-BA (218.8 F/g). Even at a high current density of 20 A/g, the specific capacitance of AHC-CL-TU reached 172.0 F/g. The cycling durability of AHC-CL-TU was investigated by conducting GCD measurements for 20,000 cycles at a charge–discharge current density of 10 A/g, as depicted in Figure 7c. AHC-CL-TU exhibited good cycling stability and a remarkable capacitance retention rate of 99.96%.

**Figure 8:**
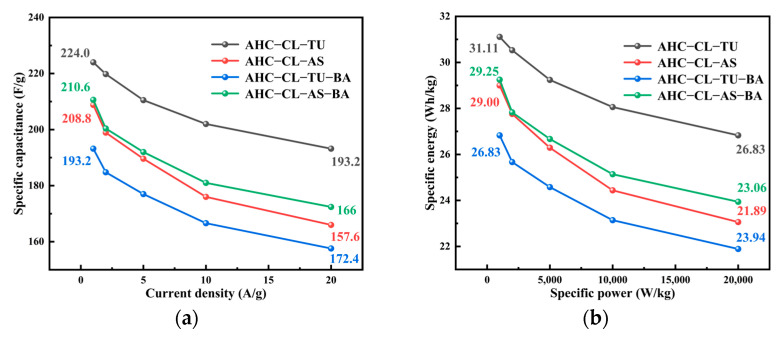
Electrochemical performance data in a two-electrode system with 6 mol/L potassium hydroxide solution as the electrolyte; (**a**) specific capacitance of AHC-CL-TU, AHC-CL-AS, AHC-CL-TU-BA, and AHC-CL-AS-BA; (**b**) energy density and power density curves for AHC-CL-TU, AHC-CL-AS, AHC-CL-TU-BA, and AHC-CL-AS-BA.

Figure 8a shows the specific capacitance data for AHC-CL-TU, AHC-CL-AS, AHC-CL-TU-BA, and AHC-CL-AS-BA determined in a two-electrode system. The results of the two- and three-electrode tests were similar, with AHC-CL-TU having the best specific capacitance. Using Formula (2), the specific capacitance was 224.0 F/g at a current density of 1 A/g, which was much higher than that of AHC-CL-AS (208.8 F/g), AHC-CL-TU-BA (193.2 F/g), and AHC-CL-AS-BA (210.6 F/g). As the current density increased, the specific capacitance of the AHC decreased slightly. Figure 8b shows the Ragone curves for AHC-CL-TU, AHC-CL-AS, AHC-CL-TU-BA, and AHC-CL-AS-BA, which showed the same trend. Using Formulas (3) and (4), the energy density and power density of this system were calculated, respectively. AHC-CL-TU provided a high energy density of 31.11 Wh/kg in the two-electrode system and a power density of 1 kW/kg at a current density of 1 A/g. It is worth noting that even if the power density was 20 kW/kg, the energy density was still as high as 26.83 Wh/kg. When the current density was 1 A/g and the power density was 1 kW/kg, the energy densities of AHC-CL-AS, AHC-CL-TU-BA, and AHC-CL-AS-BA were 29.00 Wh/kg, 26.83 Wh/kg, and 29.25 Wh/kg, respectively.

Table 3 compares the properties of N-doped carbon materials prepared by different methods. Zhao et al. prepared a B,N-codoped porous carbon via the carbonization of freeze-dried chitosan-boric acid aerogel beads. This B,N-codoped porous carbon showed a specific capacitance of 217 F/g at a current density of 1 A/g. However, the energy density of this B,N-codoped porous carbon was only 7.2 Wh/kg at a power density of 4.984 kW/kg [44]. Shan et al. prepared a N-doped carbon material via direct HTC of cellulose and ammonium sulfate, followed by KOH activation. This N-doped carbon material presented a specific capacitance of 227.3 F/g at a current density of 1 A/g. However, after 10,000 charge–discharge cycles, the capacitance retention rate of this N-doped carbon material was only 76.55% [46]. Djandja synthesized an N-doped carbon material by HTC of cellulose and pyridine. The specific capacitance of this N-doped carbon material was 214.1 F/g at a current density of 1 A/g. The capacitance retention rate was 97.82% after 20,000 charge–discharge cycles at a current density of 10 A/g [47]. In contrast, for the present study, co-HTC of cellulose and thiourea produced N-doped carbon with a specific capacitance of 235.8 F/g at a current density of 1 A/g. The capacitance retention rate remained at 99.96% after 20,000 charge and discharge cycles at a high current density of 10 A/g. The energy density reached 29.24 Wh/kg at a power density of 0.5 kW/kg.

This study addressed the issue of low charge–discharge cycling retention rates and low specific capacitances in supercapacitor carbon materials doped with heteroatoms. Nevertheless, the specific capacitance of the material remained relatively low. This study examined the impact of boric acid on various nitrogen sources during the production of polyatomically-doped supercapacitor carbon materials through hydrothermal carbonization. The results showed that boric acid enhanced the specific capacitance and retention capacity of the supercapacitor carbon. These findings offer a facile approach to synthesizing sustainable and conductive polyatomic-doped supercapacitor carbon materials.

## 4. Conclusions

Nitrogen and sulfur can be doped into cellulose during hydrothermal carbonization. The doping effects of organic nitrogen and sulfur compounds were superior to those of inorganic compounds. Boric acid exerted a negative impact on the carbon materials obtained through the cocarbonization of nitrogen sources and cellulose. This effect was particularly pronounced when thiourea was used as the organic nitrogen source. Boric acid reduced the specific surface areas of the porous materials and hindered micropore formation. It significantly impaired the electrochemical performance of the thiourea sample, but this negative effect was eliminated by cycling. During the carbonization experiments, codoping with nitrogen and boron showed that boric acid had a more pronounced inhibitory effect. Compared to AHC-CL-AS (829.59 m^2^/g) and AHC-CL-TU (952.27 m^2^/g), the specific surface areas of AHC-CL-AS-BA (484.76 m^2^/g) and AHC-CL-TU-BA (397.59 m^2^/g) were significantly lower. At a current density of 1 A/g, there was no significant difference in the specific capacitances of AHC-CL-AS-BA (219.3 F/g) and AHC-CL-AS (216.4 F/g), while that of AHC-CL-TU-BA (205.8 F/g) was significantly lower than that of AHC-CL-TU (236.25 F/g). Thus, boric acid strongly inhibited the nitrogen-doped carbonization experiments. Nonetheless, we believe that the desirable properties of supercapacitor carbon materials can be achieved through cellulose hydrothermal carbonization with nitrogen and sulfur compounds.

## Figures and Tables

**Table 1:** Elemental analyses (wt%) of HC and AHC.

Samples Name	C	H	N	S	O	H/C	O/C
CL	66.61	4.44	0	0	28.55	0.07	0.43
HC-CL	70.14	4.35	0	0	24.07	0.06	0.34
HC-CL-AS	69.98	5.12	3.63	0.35	19.36	0.07	0.28
HC-CL-TU	67.34	6.20	4.67	2.92	17.94	0.09	0.27
HC-CL-AS-BA	63.21	4.66	4.92	4.54	20.81	0.07	0.33
HC-CL-TU-BA	69.54	4.81	4.31	0.37	20.14	0.07	0.29
AHC-CL	76.11	1.27	0	0	21.91	0.02	0.29
AHC-CL-AS	79.59	1.88	3.84	0.32	12.69	0.02	0.16
AHC-CL-TU	78.32	1.94	3.25	2.23	12.46	0.02	0.16
AHC-CL-AS-BA	78.26	1.74	4.02	3.97	11.27	0.02	0.14
AHC-CL-TU-BA	79.34	1.79	3.97	0.32	12.73	0.02	0.16

**Table 2:** XPS-derived elemental contents (wt%) of the AHC.

Sample Names	C1s	N1s	S2p	O1s	B1s
AHC-CL-AS	72.60	2.54	0.72	24.14	
AHC-CL-TU	68.91	3.2	3.73	24.16	
AHC-CL-AS-BA	68.98	3.33	4.10	23.19	0.39
AHC-CL-TU-BA	70.49	4.33	0.93	23.44	0.80

**Table 3:** Comparison of the properties of N-doped carbon materials prepared by different methods.

Raw Materials and Preparation Methods	Advantage	Electrochemical Properties
Carbonization of freeze-drying chitosan-boric acid aerogel beads [44]	High performance; multiple atomic doping	The specific capacitance was 217 F/g at a current density of 1 A/g. The capacitance retention rate was 95.1% after 10,000 cycles of charge and discharge at a current density of 5 A/g. Energy density was 7.2 Wh/kg at a power density of 4.984 kW/kg.
Co-HTC of cellulose and ammonium sulfate [46]	High specific surface area; multiple atomic doping	The specific capacitance at 1 A/g current density was 227.3 F/g, the capacitance retention rate at 20 A/g current density was 76.55%.
Co-HTC of cellulose and pyridine [47]	High capacitance retention; two-stage HTC	The specific capacitance is 214.1 F/g at a current density of 1 A/g. After 10,000 charge–discharge cycles at a current density of 5 A/g, the capacitance retention rate is 97.82%.
Co-HTC of cellulose and thiourea (This study)	High specific capacitance; high cycle retention rate; simple process	The specific capacitance reached 235.8 F/g at a current density of 1 A/g. After 20,000 charge and discharge cycles at a high current density of 10 A/g, the capacitance retention rate was 99.96%. Energy density was 29.24 Wh/kg at a power density of 0.5 kW/kg.

## Data Availability

The data presented in this study are available upon request from the corresponding author.
